# Prevalence, Safety, and Metabolic Control Among Danish Children and Adolescents with Type 1 Diabetes Using Open-Source Automated Insulin Delivery Systems

**DOI:** 10.1089/dia.2023.0412

**Published:** 2024-04-30

**Authors:** Amanda R. Fagerberg, Luise Borch, Kurt Kristensen, Jesper S. Hjelle

**Affiliations:** ^1^ Department of Pediatrics and Adolescent Medicine, Goedstrup Regional Hospital, Herning, Denmark.; ^2^ Steno Diabetes Center Aarhus, Aarhus Univeristy Hospital, Aarhus, Denmark.

**Keywords:** Artificial pancreas, Closed-loop systems, Pediatrics, Type 1 diabetes, Time in range, HbA1c, Do-It-Yourself Closed-Loop Systems, Open source, Sleep

## Abstract

**Background:**

Treatment of type 1 diabetes mellitus (T1DM) has become increasingly technical with rapid developments in integration of pumps and sensors to regulate insulin dosage, and patient-initiated solutions as open-source automated insulin delivery (OS-AID) systems, have gained popularity in people with diabetes. Studies have shown increased glycemic control and mental wellbeing in users of OS-AID systems. The aim of this study was to estimate the prevalence, the effect on metabolic control, the risk, and the effect on everyday life for users and their parents of OS-AID systems in Danish children and adolescents with T1DM.

**Methods:**

This retrospective cohort study recruited participants through pediatric diabetes outpatient clinics and social media. Surveys were distributed and current and retrospective data on glycemic control (HbA1c, time in range [TIR] etc.) were collected.

**Results:**

Fifty-six users of OS-AID systems out of 2950 Danish children and adolescents with T1DM were identified from all outpatient clinics in Denmark. Thirty-one responded on contact and were included (55% of the identified), median age 12 [interquartile range: 11–14] years, 51% females, and mean duration of use of OS-AID systems 2.37 ± 0.86 years. Glycemic control increased significantly with TIR increasing from mean 62.29% ± 13.68% to 70.12% ± 10.08%, *P < 0.01, and HbA1c decreasing from mean 50.13 ± 5.76 mmol/mol (6.7% ± 2.7%) to 47.86 ± 6.24 mmol/mol (6.5% ± 2.7%), **P < 0.05. No changes were found in safety parameters. Parents reported better quality of sleep evaluated by Pittsburgh Sleep Quality Index.

**Conclusion:**

This study is the first to provide knowledge on pediatric users of OS-AID systems in Denmark and found a prevalence of 1.89% for OS-AID systems, improved TIR, and no increased risk associated with use of OS-AID systems.

## Introduction

Type 1 diabetes mellitus (T1DM) is an autoimmune chronic condition and people with diabetes (PwD) need to constantly monitor their blood glucose (BG) and administer exogenous insulin to meet treatment goals.^1,2^ These are important to meet as dysregulated T1DM can lead to long-term complications such as neuropathy, retinopathy, nephropathy, or acute life-threatening complications such as severe hypoglycemia or diabetic ketoacidosis.^3,4^ In Denmark in 2021–2022, 2950 children and adolescents <18 years of age lived with the diagnosis T1DM.^5^

Treatment goals for metabolic control in children and adolescents with T1DM are by “ISPAD Consensus Guidelines 2022” defined as HbA1c <53 mmol/mol (<7.0%).^6^ Other measurements for monitoring treatment include percentage of time spent with glucose in different ranges (time in range [TIR], time below range [TBR], and time above range [TAR]), as well as glucose variability measured as coefficient of variation (CV) to assess fluctuations in BG.^7,8^ Psychosocial factors can influence the metabolic regulation of children and adolescents with T1DM, including socioeconomic status.^9^ Higher socioeconomic status in parents of children and adolescents with T1DM is associated with better diabetes management.^10^

Treatment of T1DM is complex and includes various options for monitoring BG and administering insulin. In recent years, capillary BG measurements and multiple daily injections of insulin have been replaced by continuous glucose monitoring (CGM), sensors, and continuous subcutaneous insulin infusion pumps.^11^ Furthermore, the development toward automated insulin delivery (AID) systems has accelerated, resulting in integration of pumps and sensors enabling automated disruption of insulin influx at low glucose levels and even enabling AID based on current BG levels and fluctuations. An AID system consists of an insulin pump, a CGM and an algorithm analyzing fluctuations in BG and adjusting insulin delivery through the pump.^12^

Concurrently with the company-based development of AID systems occurred, a group of PwD initiated the development of open-source automated insulin delivery (OS-AID) systems, using the hashtag #WeAreNotWaiting.^13^ Even though commercial AID systems are available, OS-AID systems continues to be of interest, as PwD still use them, and not all diagnosed with T1DM in Denmark are prescribed the new commercial AID systems.

Two OS-AID algorithms are developed, OpenAPS and Loop iOS. AndroidAPS and iAPS have been developed based on the OpenAPS algortihm.^11,14–17^ All available AID systems require some level of technical abilities from the user and OS-AID systems are no exception. OS-AID software must be installed on a hardware either by following detailed manuals online or by getting help from other users/programmers. Furthermore, maintenance and problem solving of the OS-AID system require technical abilities and courage as there are no customer service to contact with technical questions, and users therefore often tend to resolve such problems with help from online communities.^14–16,18^ None of the OS-AID systems available is approved by Danish health authorities, thus leaving health care professionals with an uncertainty on how to advise people who chooses to use an OS-AID system.^19,20^

In addition, the responsibility for metabolic control to a greater extend lies with PwD themselves when using OS-AID systems compared to authorized medical technology, as health care professionals cannot advise their patients on the installation of OS-AID systems, although they still can advise on the treatment of diabetes according to guidelines from the Danish Steno Diabetes Center Copenhagen.^21^

The number of international studies investigating OS-AID systems is growing and include retrospective and prospective studies using real-world data and in addition randomized clinical trials. The studies found increased glycemic control with decrease in HbA1c, increase in TIR as well as increased quality of life for the users or their potential caregivers.^22–26^

To our knowledge no studies have investigated nationwide pediatric use of OS-AID systems. This leaves PwD, health care professionals, and caregivers with a shortage of knowledge when making treatment decisions or advising patients.

This study aims to investigate the nationwide prevalence, safety, and metabolic effect of OS-AID systems in Danish children and adolescents with T1DM as well as the effect on everyday life for users and their parents.

## Materials and Methods

The study is an observational study with data collected from data platforms and patient records as well as surveys and psychometric questionnaires. The study was approved by the Central Denmark Region Comity of Medical research, and the approval applied for the entire country.

### Inclusion of participants

Participants were identified from all pediatric diabetes outpatient clinics in Denmark and through announcements in two Danish Facebook groups: “Looped - Denmark” and “Parents of children with type 1 Diabetes.” Outpatient clinics were asked to count children and adolescents aged 2–18 in their clinic using OS-AID systems and distribute information about the study. Parents or legal representatives signed electronic consent forms. Enrollment was open from December 2021 to December 2022. Inclusion criteria were age 2–18 years, T1DM, and use of an OS-AID system at time of enrollment. Fifteen pediatric clinics are treating pediatric diabetes patients in Denmark and the number of patients treated varied from 64 to 699 at the time of this study.^5^

### Data collection

To assess prevalence, outpatient clinics for pediatric diabetes were asked to report the total number of persons aged 2–18 using OS-AID systems at their clinic. This information was combined with data from The Danish Registry of Childhood and Adolescent Diabetes on the total number of T1DM diagnosed children and adolescents in Denmark.^5^

Baseline was defined as the period before initiation of an OS-AID system. Glycemic data were collected retrospectively from patient records and the data platforms Diasend^®^, Glooko^®^, Nightscout^®^, and Tidepool^®^. From data platforms, the following data were collected: percentage of time spent with glucose in TIR 3.9–10 mmol/L (70–180 mg/dL), TBR <3.9 mmol/L (<79 mg/dL), TAR >10 mmol/L (>180 mg/dL), and mean BG and CV.^8^ Baseline data were collected from the 4-week period before initiating an OS-AID system, and data from after initiation of an OS-AID system were collected from 1–8 weeks, 9–16 weeks, and 17–24 weeks after initiation as well as the latest available 4-week period.

From patient records, HbA1c, weight, and height 6 months before and 6 months after initiating an OS-AID system, as well as the latest available values were collected. Had the participant used an OS-AID system for only 6 months or less at the time of data collection, then latest available data within the 6 months were used as latest available values. HbA1c measurements were from venous or capillary blood, depending on the data available from the individual, although consistent within the individual participant.^27^ Furthermore, information about ketoacidosis and severe hypoglycemia 1 year before and 1 year after initiation of an OS-AID system were recorded. Severe hypoglycemia was defined as BG <3.9 mmol/L and one or more of the following: having experienced unconsciousness or seizure or being disoriented or incoherent compared to normal state.^28^

Ketoacidosis was defined as contact with the hospital and the following criteria: hyperglycemia >11 mmol/L, venous pH <7.3 or serum bicarbonate <15 mmol/L, and ketonemia with ß-hydroxybutyrate >3 mmol/L.^4^ Diabetic ketoacidosis at diabetes debut was excluded. BMI (body mass index) was calculated using data on weight and height from 6 months prior, 6 months after, and latest available data. BMISDS (body mass index standard deviation score) was calculated using the Danish pediatric BMI chart on healthy individuals as reference.^29,30^

### Questionnaires

At the time of enrollment, a survey regarding demographics, diabetes diagnosis, medical history, experience of using the OS-AID system, socioeconomic status, family, and selected psychometric questionnaires were distributed. The psychometric questionnaires used were Pittsburgh Sleep Quality Index (PSQI), Self-Efficacy in Diabetes Management (SEDM), WHO-5, and Hypoglycemia Fear Survey Worry Subscale (HFS) (Supplementary Table S1).^31–37^ PSQI was taken twice per respondent: once recollecting parent's sleep quality retrospectively, before their child initiated an OS-AID system, and once regarding parent's sleep-quality at enrollment. Likewise, SEDM was taken twice per respondent: once regarding the participant's self-efficacy retrospectively, before initiating an OS-AID system, and once regarding their self-efficacy at enrollment. Both participants and parents of participants responded to the WHO-5 questionnaire and the HFS questionnaire.

Both WHO-5 and HFS were taken once per respondent regarding well-being and fear of hypoglycemia, respectively, at enrollment (Supplementary Table S1). WHO-5, PSQI, and SEDM are validated in Danish, and HFS was translated for this study.^31,33–35,37,38^ Translation was performed with two forward translators fluent in Danish and English with knowledge of the diabetes field, their Danish translations were compared, and then a backward translation was performed by a professional translator. The Danish version was tested by two adolescents with T1DM and their parents. All data were collected and managed using REDCap (Research Electronic Data Capture) hosted at Aarhus University.^39,40^

### Statistical methods

Statistical analysis was performed in STATA version 17.0 (StataCorp LLC, College Station, TX, USA).^41^ For descriptive statistics, variables were reported as mean ± standard deviation (SD) when data were normally distributed or with median and interquartile range [IQR] when data were not normally distributed. Unless otherwise stated, data are noted as mean *±* SD. To compare outcomes pre- and postinitiation of OS-AID systems, paired *t*-test was used, and outcome reported as mean [95% CI] when differences in data were normally distributed. When difference in data were skewed, a Wilcoxon signed rank test was used instead. Statistical significance was defined as a two-tailed *P* < 0.05.

Primary outcomes were prespecified as prevalence and changes in TIR at baseline compared to TIR after initiation of OS-AID systems. Secondary outcomes were prespecified as HbA1c, percentage of TAR 10.1–13,9 mmol/L (180–250 mg/dL), TAR >13.9 mmol/L (>250 mg/dL), TBR 3.0–3.8 mmol/L (54–70 mg/dL), TBR <3.0 mmol/L (>54 mg/dL), number of diabetic ketoacidosis and severe hypoglycemia, mean BG, CV, outcomes for WHO-5, HFS-C, HFS-P and HFS-PYC Worry Subscale, changes in SEDM, and PSQI scores from the respond regarding baseline to the respond regarding the current period.

## Results

### Participant characteristics

All pediatric diabetes outpatient clinics kindly contributed and reported the number of users of OS-AID systems treated at their clinic, identifying 56 users. Thirty-one responded and were included in the study. Of the 31 included, 3 participants did not answer the survey, data on Hba1c were missing in 4 participants, data on TIR from data platforms were missing in 10 participants and data on height and weight were missing in 10 participants. Of the 31 included, 15 were male (48%) and 16 females (52%). Participants were 12 [11–14] (median [IQR]) years old and had been using OS-AID systems for 2.37 ± 0.86 years. Of the 28 participants who answered the survey, all used an Omnipod Eros or Dash insulin pumps, 27 used Dexcom CGM and 1 used Libre CGM. Twenty-five used Loop iOS, 2 used Android APS, and 1 used OpenAPS for OS-AID algorithm.

At baseline, all participants used insulin pump and CGM without an OS-AID system or other AID systems, except one participant who used a commercial AID system before initiating the OS-AID system. In only one participant, the primary responsibility regarding the use and settings of the OS-AID system laid with the child or adolescent itself. In all other participants, the primary responsibility lay with adult caregivers. Participants reported usage of “Profiles” with altered BG targets daily (21%), weekly (46%), monthly (11%), less than six times per year (4%), and never (18%). Respondents were asked to report the mother's education at birth of the participant, and they reported high school (3%), vocational or 2-year college (25%), bachelors (36%), or masters (36%). For further characteristics of the participants, see Supplementary Table S2.

### Prevalence of OS-AID systems among Danish children and adolescents with T1DM

In the period of March to May 2022, 56 persons with T1DM aged 2–18 used OS-AID systems in Denmark, when compared to the total number of children and adolescents with T1DM (2950) registered 2021–2022, this corresponds to 1.89% of the pediatric population of T1DM using OS-AID systems.

### Glycemic effect of OS-AID systems

**FIG. 1.**
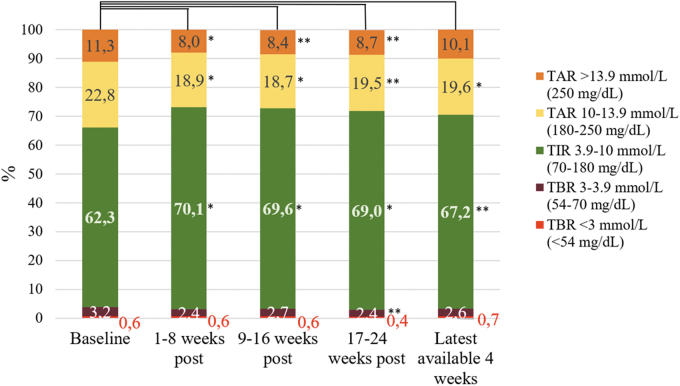
Showing percentage of time spent with BG in the following ranges: TIR 3.9–10.0 mmol/L (70–180 mg/dL); TAR 10.1–13.9 mmol/L (180–250 mg/dL); TAR >13.9 mmol/L (>250 mg/dL); TBR 3.0–3.9 mmol/L (54–70 mg/dL); TBR <3.0 mmol/L (<54 mg/dL). *Significant changes in percentage of time spent in the specific range when compared to baseline with *P*-value <0.01; **Significant changes in percentage of time spent in the specific range when compared to baseline with *P*-value <0.05. TAR, time above range; TBR, time below range; TIR, time in range.

CGM data were available in 21 participants. TIR increased significantly from mean 62.29% ± 13.68% at baseline to mean 70.12% *±* 10.08%, *P < 0.01, in the first 8 weeks after initiation of OS-AID systems (Table 1 and Fig. 1). The significant increase in TIR persisted throughout the 24 weeks after initiation of OS-AID systems and in the latest available 4 weeks of CGM data (Table 1 and Fig. 1). A significant decrease in TAR was found from mean 22.80% *±* 5.96% at baseline to mean 18.91% ± 5.42%, *P < 0.01, after initiation of OS-AID systems and persisted throughout the 24 weeks after initiation and as well in the latest available 4 weeks of CGM data (Table 1 and Fig. 1).

**Table 1.** Percentage of Time in Range, Time Above Range, Time Below Range, Average Blood Glucose, and Coefficient of Variation from Continuous Glucose Monitoring Data

*n* = 21	Before OS-AID systems	After OS-AID systems			
	4 Weeks before (baseline)	1–8 Weeks after	9–16 Weeks after	17–24 Weeks after	Latest available 4 weeks
TIR 3.9–10.0 mmol/L in %, mean ± SD	62.29 *±* 13.68	70.46 *±* 11.56	69.55 *±* 10.93	69.02 *±* 11.06	67.21 *±* 12.13
Difference from baseline, mean [95% CI]		7.83 [4.12 to 11.54]^*^	7.25 [3.07 to 11.43]^*^	6.72 [2.38 to 11.07]^*^	5.91 [1.14 to 8.69]^**^
TAR 10.0–13.9 mmol/L in %, mean ± SD	22.80 ± 5.96	18.91 ± 5.42	18.71 ± 5.05	19.46 ± 4.79	19.55 ± 5.16
Difference from baseline, mean [95% CI]		−3.88 [−6.47 to −1.30]^*^	−4.08 [−6.67 to −1.49]^*^	−3.33 [−6.21 to −0.45]^**^	−3.24 [−5.49 to −0.98]^*^
TAR >13.9 mmol/L in %, mean ± SD	11.33 ± 8.81	7.98 ± 6.02	8.41 ± 8.37	8.66 ± 8.70	10.06 ± 9.69
Difference from baseline, mean [95% CI]		−3.35 [−5.39 to −1.31]^*^	−2.91 [−5.23 to −0.59]^**^	−2.66 [−4.69 to −0.64]^**^	−1.27 [−3.78 to 1.24]
TBR 3.0–3.8 mmol/L in %, mean ± SD	3.22 ± 2.39	2.41 ± 2.19	2.68 ± 2.51	2.40 ± 2.04	2.61 ± 2.15
Difference from baseline, mean [95% CI]		−0.81 [−1.74 to 0.12]	−0.54 [−1.43 to 0.35]	−0.81 [−1.56 to 0.07]^**^	−0.60 [−1.55 to 0.33]
TBR <3.0 mmol/L in %, mean ± SD	0.58 ± 0.86	0.57 ± 1.20	0.56 ± 0.95	0.40 ± 0.62	0.65 ± 0.82
Difference from baseline, mean [95% CI]		−0.00 [−0.53 to 0.53]	−0.01 [−0.43 to 0.40]	−0.17 [−0.43 to 0.08]	0.07 [−0.35 to 0.49]
Average BG in mmol/L (mg/dL), mean ± SD	9.00 ± 1.16 (162.2 ± 20.90)	8.39 ± 1.01 (151.17 ± 18.20)	8.44 ± 1.26 (152.07 ± 22.70)	8.59 ± 1.23 (154.77 ± 22.16)	8.72 ± 1.40 (157.12 ± 25.23)
Difference from baseline, mean [95% CI]		−0.60 [−0.99 to −0.21]^*^ (−10.81 [−17.84 to −3.78])	−0.55 [−0.96 to −0.15]^*^ (−9.91 [−17.30 to −2.70])	−0.40 [−0.76 to −0.05]^**^ (−7.21 [−13.69 to −0.90])	−0.27 [−0.71 to 0.16] (−4.86 [−12.79 to 2.88])
CV in %, n = 20, mean ± SD	39.05 ± 5.25	38.42 ± 5.29	38.23 ± 4.37	37.77 ± 4.60	39.22 ± 5.80
Difference from baseline, mean [95% CI]		−0.62 [−2.53 to 1.27]	−0.81 [−2.61 to 0.99]	−1.27 [−2.95 to 0.41]	−0.17 [−1.98 to 2.33]

AID, automated insulin delivery; BG, blood glucose; CI, confidence interval; CV, coefficient of blood glucose variation; OS-AID, Open-Source Automated Insulin Delivery; SD, standard deviation; TAR, time above range; TBR, time below range; TIR, time in range.

^*^ *P*-value <0.01; ^**^P-value <0.05.

TAR >13.9 mmol/L (>250 mg/dL) decreased significantly in the 24 weeks after initiation of OS-AID systems compared to baseline, but the decrease did not persist in the latest available 4 weeks of CGM data. Average BG decreased from baseline to the first 8 weeks after initiation of OS-AID systems from mean 9.00 ± 1.16 mmol/L (162.20 ± 20.90 mg/dL) to 8.39 ± 1.01 mmol/L (151.17 ± 18.20 mmol/L), *P < 0.01. This significant decrease persisted throughout the first 24 weeks following initiation of OS-AID systems but did not persist in the latest available 4 weeks of CGM data. No change was seen in CV (Table 1). Mean time from initiation of OS-AID systems to the last date of the latest available 4 weeks period of CGM data were 618 [391–835] days (median [IQR]).

HbA1c data were available in 27 participants. A significant decrease was seen from baseline mean 50.13 ± 5.76 mmol/mol (6.7% ± 2.7%) to mean 47.86 ± 6.24 mmol/mol (6.5% ± 2.7%), **P < 0.05, 0–6 months after initiation of OS-AID systems. No significant decrease was seen when baseline was compared to the latest available HbA1c: difference −2.28 [95% CI: −5.31 to 0.73] mmol/mol, from mean 50.13% ± 5.76 mmol/mol (6.7% ± 2.7%) to mean 47.85 ± 7.44 mmol/mol (6.57 ± 2.8%), *P* = 0.13. Mean time from initiation of OS-AID systems to latest available HbA1c was 515 [308–623] days (median [IQR]). Data on weight and height were available in 21 of the participants. BMISDS increased significantly from baseline compared to the latest available data after initiation of OS-AID systems: from mean − 0.00 ± 1.18 to mean 0.28 ± 1.28 (difference: 0.28 [95% CI: 0.07–0.50]), **P < 0.05.

Time from baseline BMISDS to latest available BMISDS was 639.5 ± 251.3 days. No significant change was seen in BMISDS in the first 6 months after initiation of OS-AID systems. No difference in BMISDS was found between genders (for more details, see Supplementary Table S3).

### Risk of OS-AID systems

Data of possible ketoacidosis and severe hypoglycemia were available in 28 participants. No diabetic ketoacidosis or incidences of severe hypoglycemia were reported 1 year before and 1 year after initiation of OS-AID systems. No significant changes were seen in TBR 3.0–3.8 mmol/L (54–70 mg/dL) or TBR <3.0 mmol/L (<54 mg/dL).

### Participants' psychometric outcomes

Twenty-six participants responded to the WHO-5 questionnaire and reported a median score of 76 [IQR: 60–80], indicating mental well-being (Table 2).

**Table 2.** Results of Administered Psychometric Questionnaires

Name of the questionnaire	No. of respondents	Characteristics of respondents	Results	Comments of results
WHO-5	26 Participants 28 Parents of participants	Median age 12.5 [IQR: 12–14] years 12 = female	Median score of 76 [IQR: 60–80] with no difference. between males and females Median score of 78 [IQR: 68–80].	Results from both parents and participants indicated mental well-being.
SEDM	20 Participants Taken twice per respondent regarding pre- and postinitiation of OS-AID systems	Mean age 14.10 *±* 1.86 years 10 = female DIY-LOOP use mean 2.29 *±* 0.84 years	Score increased from mean 68.55 *±* 14.56 at baseline *compared to* mean 82.15 *±* 14.95, *P* < 0.01^*^ No difference between males/females	Results indicated an increase in self-efficacy.
HFS-C HFS-P	22 Participants 26 Parents of children aged 8–17	Mean age 13.90 *±* 1.87 years 11 = female	Mean score of 7.77 *±* 4.58 No difference between males/females Mean score of 14.7 *±* 8.57	Results indicated more worry in parents than participants concerning hypoglycemic events. Neither the parents' nor participants' scores indicated excessive worry.
PSQI	25 Parents of participants Taken twice per respondent regarding pre- and postinitiation of OS-AID systems		A decrease in the score from mean 8.64 *±* 3.9 at baseline *compared to* mean 3.68 *±* 2.59, *P* < 0.01^*^ after initiation of OS-AID systems	Results indicated an increase in sleep quality

HFS-C, Hypoglycemia Fear Survey Children; HFS-P, Hypoglycemia Fear Survey Parents; HFS-PYC, Hypoglycemia Fear Survey Parents of Young Children; IQR, interquartile range; OS-AID, Open-Source Automated Insulin Delivery; PSQI, Pittsburgh Sleep Quality Index; SEDM, Self Efficacy in Diabetes Management;

Twenty adolescent participants responded to the SEDM psychometric questionnaire, and the score increased from mean 68.55 *±* 14.56 at baseline *compared to* mean 82.15 *±* 14.95, *P < 0.01, indicating an increase in self-efficacy (Table 2).

Twenty-two participants responded to the HFS-C Worry Subscale and reported a mean score of 7.77 *±* 4.58. This did not indicate excessive worry (Table 2).

### Parents' psychometric outcomes

Parents of 25 participants responded to the PSQI questionnaire, and the score decreased from mean 8.64 *±* 3.9 at baseline *compared to* mean 3.68 *±* 2.59, *P < 0.01, indicating an increase in sleep quality (Table 2).

Parents of 28 participants responded to the WHO-5 psychometric questionnaire and reported a median score of 78 [IQR: 68–80] indicating mental well-being (Table 2).

Twenty-six parents of children aged 8–17 responded to the HFS-P Worry Subscale and reported a mean score of 14.7 *±* 8.57. This did not indicate excessive worry although the score indicates more worry in parents than participants (Table 2).

## Discussion

To the best of our knowledge, this study is the first investigating the prevalence, safety, and metabolic control of OS-AID systems in a whole nation. The prevalence of OS-AID systems is 1.89% among the Danish pediatric population of PwD in spring 2022. In The Danish Registry of Childhood and Adolescent Diabetes information on use of pumps and sensors are found in 2311 of 2950 children and adolescents with T1DM. Of these 2311, 79.70% use any type of pump (1842/2311) and 92.55% (2139/2311) use sensor more than 70% of the time.^5^ The group of pediatric users of OS-AID systems is thus a small subgroup. Prevalence data were based upon health care providers reports from outpatient clinics. It is argued that the outpatient clinics are aware of the few users of OS-AID systems at their clinic, and therefore the estimate of prevalence is regarded valid.

This present study also provides evidence of increased glycemic control with use of OS-AID systems. A significant increase in TIR in the 24 weeks following initiation of OS-AID systems and in the latest available 4 weeks period of CGM data was seen with greatest effect on higher glucose levels. Furthermore, a significant decrease in HbA1c was found in the 24 weeks following initiation of OS-AID systems. This study did not show any significant effect on HbA1c in the latest available 4 weeks period of CGM data, although a tendency toward lower HbA1c appeared. It can be argued that the effect on HbA1c is masked by the known increase in HbA1c during adolescent years.^42^

It is noteworthy, that even though the participants present at baseline with average HbA1c of 50.13 ± 5.76 mmol/mol (6.7% ± 2.7%), and therefore meet treatment goals defined as HbA1c <53 mmol/mol (< 7.0%) by “ISPAD Consensus Guidelines 2022” *before* initiating OS-AID systems, they still benefit from OS-AID systems with an increased glycemic control.^27^ The clinical significance of this HbA1c effect can although be discussed in these otherwise well-regulated PwD, the metabolic effects of initiating OS-AID systems is noninferior, and in addition adds other effects to the user's daily life and decreases overall burden.

No increased risk was seen regarding the number of ketoacidosis, incidences of severe hypoglycemia, or TBR. BMISDS increased significantly from baseline to latest available BMI. It is known that higher insulin dose is correlated with higher BMISDS and it could be speculated if this is the case as well with OS-AID systems.^43^ It is noteworthy that the BMISDS in this study is lower than found in other studies investigating children and adolescents with T1DM.^43^ This could be due to the fact, that the users of OS-AID systems have a socioeconomic status above average. Studies investigating commercial AID systems have found no change in BMISDS after 6 months and 1 year.^44,45^ In this study, the change in BMISDS was seen later than 1 year after initiation of OS-AID systems.

Results from the present study support the findings of other retrospective studies: Petruzelkova et al. investigated 36 Czech children and adolescents and found a reduction in HbA1c in children aged 8–16 from 52.6–45.1 mmol/mol (7%–6.3%) and an increase in TIR of 77.2%–82.9%.^46^ Braune et al. investigated 209 children and adolescents from 21 different countries and found a reduction in HbA1c from 52 to 45 mmol/mol (6.91%–6.27%) and an increase in TIR from 64.2% to 80.68%.^25^ Lum et al. investigated users of Loop iOS prospectively over 6 months in a large cohort of 558 children and adults where 169 were aged 7 to <14. All were United States residents. In the 7 to <14 years of age an increase in TIR from 64% to 69% was seen as well as a decrease in Hba1c from 53 to 49 mmol/mol (7%–6.6%).^24^

The study by Petruzolkova et al. is similar to the present study regarding population size but is not assessing HbA1c beyond the 24 weeks following initiation of OS-AID systems, and is only assessing users of AndroidAPS, whereas participants of the present study mainly use Loop iOS.^46^ Braune et al. is assessing a greater population size compared to the present study but is only assessing TIR and HbA1c.^25^ Both Petruzelkova et al. and Braune et al. are assessing glycemic effect based on self-reported data from participants. Lum et al. are assessing a greater population size than the present study, are collecting data from data platforms, and are only investigating users of Loop iOS which reflects the algorithms used by participants of the present study.^24^

The present study shows strength as the glycemic data are collected from data platforms and patient records rather than self-reported by participants. On the contrary, the participant number in the present study is small compared to Braune et al. and Lum et al., which could bias the results by under- or overestimating the effect of OS-AID systems.

As this study did not aim to compare with the commercial AID systems, the effects on TIR, HbA1c, and so on are not to be compared, even though real-life data on these commercial AID systems report greater effects than in this present study.^47–49^ Other studies aiming specifically at comparing OS-AID systems with commercial AID systems found OS-AID systems to be non-inferior in adults and in real-life data on children, the same was found.^50,51^

The educational level of mothers at the time of birth of the participant are higher when compared to mothers of all Danish children and adolescents with T1DM as shown in Nielsen et al.^10^ These findings, in addition with the high occupational rate of mothers, indicates that pediatric users of OS-AID systems come from families with a socioeconomic status above average. A similar pattern of socioeconomic factors among users of OS-AID systems has been seen in other studies.^24,25^ It could be speculated, that parents with higher education are more likely to seek out information of OS-AID systems and can process this knowledge to a greater extent than less educated parents.

As it is known, socioeconomic status can affect HbA1c and could therefore be considered a confounder which could affect glycemic results to be more evident. This confounder is diminished in the present study, as the participants are used as their own controls. When assessing the effect of OS-AID systems on everyday life, the present study found an increase in quality of sleep for parents and an increase in self-efficacy for adolescent participants. Both changes are grounded in the respondents' memory of the period preceding the initiation of the OS-AID system, and therefore an uncertainty due to recall bias must be considered. To still take these findings into consideration, it could be argued that the measurement wished to assess is the experienced change.

The changes experienced in self-efficacy during adolescent years should also be assessed with skepticism, as they could arise from confounders such as greater adaptation to responsibilities following T1DM as well as an increase in age, which could overestimate the effect of OS-AID systems on self-efficacy. Furthermore, the present study finds a high level of mental well-being among both parents and participants. Both the findings on parents' sleep quality and parents' mental well-being are supported by findings in the study by Knoll et al. which finds WHO-5 score of 65.5 *±* 19.8 (*n* = 101) and a PSQI score of 5.0 ± 3.1 (*n* = 90) in parents of users of OS-AID systems.^52^

Limitations of this study is to be considered, as the populations' size is relatively small, even though it reflects the national population size of pediatric users of OS-AID systems. In addition, only 31 of the known 56 Danish pediatric users of OS-AID systems are included in this study. This could bias the results, as it is uncertain why some users did not wish to be included. It could be speculated, that users not wanting to participate may have worse glycemic outcomes, and therefore this inclusion bias could possibly overestimate the effect of OS-AID systems on glycemic outcomes. It was not possible to collect data on users who did not want to participate, except no geographical tendency was found, as participants were included from all outpatient clinics reporting to treat users of OS-AID systems regardless of the clinic being in an urban or rural area.

Likewise, the lack of inclusion of users who have discontinued the use of OS-AID systems could bias the glycemic results toward a greater outcome, as this study only assesses participants still using an OS-AID system at enrollment. Finally, it is important to note that results of this study are not generalizable as the participants were generally well regulated even before initiating OS-AID systems. Furthermore, there are indicators that participants differ regarding socioeconomic status when compared to the general Danish pediatric population with T1DM, which again weakens the generalizability of the results of this study.

The strengths of the present study are to be taken into consideration as well. First, data are collected from patient records and data platforms rather than by self-reported data. This strengthens the reliability of the results, as the possibility of bias regarding reporting of glycemic measurements is low. Furthermore, this study includes all pediatric outpatient clinics in Denmark and therefore represents the whole country. Next, the effect on glycemic control is thoroughly investigated, as several glycemic parameters are taken into consideration such as HbA1c and TIR, TBR, and TAR. At last, this study is the first to assess the Danish national prevalence of OS-AID systems among the pediatric population with T1DM.

With new commercially available AID systems on the market, the role of OS-AID systems is changing. Will people with T1DM still initiate use of OS-AID systems or would they rather use commercial AID systems? As studies show similar glycemic results when comparing OS-AID systems and commercially available AID systems,^22^ it can be argued that the choice will be based on other qualities. Such qualities could be the higher degree of individualized settings in OS-AID systems due to liability issues in the commercial AID systems, which might be an argument for some to initiate OS-AID systems rather than commercially available AID systems. On the contrary, the possibility to get help from companies regarding technical issues might be an argument for others to choose the commercially available AID systems. Furthermore, financial cost could influence the future role of OS-AID systems.

In Denmark, commercial AID systems are distributed for free to most children and adolescents with T1DM, and therefore cost should not influence the choice of insulin delivery system. In countries where access to commercial AID systems is not funded by public health insurance, OS-AID systems might offer a cheaper alternative, which could be an incentive to use the latter. Finally, there is, however, a risk that the pumps compatible in an OS-AID system will be taken off the market, as the companies supplying them will have their own AID systems, and therefore have no incentive to keeping the older models available on market. Either way, we do, however, argue, that due to the fast development within the OS-AID community as no approval is needed, the OS-AID community will continue to influence the development within diabetes technology in the future.

As this present study shows better glycemic control but also a small subgroup of users with higher socioeconomic status, the data are not strong enough to recommend the use of OS-AID systems to all Danish children and adolescents with T1DM as the results are not generalizable to the general pediatric T1DM population. Nonetheless, the present study gives an important knowledge on the subgroup who are already using OS-AID systems by their own initiative.

## Conclusion

This study is the first to assess a nationwide pediatric population with T1DM using OS-AID systems. We found a prevalence of OS-AID systems of 1.89% among Danish children and adolescents diagnosed with T1DM. Users of OS-AID systems had a significant increase in glycemic control following initiation of OS-AID systems, and no increased risk of ketoacidosis or hypoglycemia.

This study provides important knowledge about the Danish pediatric diabetes population who uses OS-AID systems. Future studies are needed to further investigate the effect of OS-AID systems in children and adolescents with T1DM. Especially, there is a need for comparison of commercial AID systems and OS-AID systems, including user characteristics to investigate the effect of these systems in people with different socioeconomic status. It would furthermore be interesting to investigate the discontinuation of OS-AID systems.

## Supplementary Material

Supplemental data
