# Supplementary material for: Prevalence, Safety, and Metabolic Control Among Danish Children and Adolescents with Type 1 Diabetes Using Open-Source Automated Insulin Delivery Systems
Source: Diabetes Technol Ther. 2024 Apr 30;26(5):287–97. doi: 10.1089/dia.2023.0412 (PMC11058411; doi:10.1089/dia.2023.0412)
Supplement: Supplemental data [file Suppl_TableS1-S3.pdf]

## Supplementary

### Suppl. Table 1

Administered Psychometric Questionnaires

| **Name of the questionnaire** | **Assessed subject** | **Administered to** | **Questionnaire answered regarding pre or post initiation of OS-AID system** | **Scoring** |
|---|---|---|---|---|
| **WHO-5** | Subjective psychological well-being. | Participants aged 9-17.<br>Parents. | Post initiation of OS-AID system . | 0-100<br><50 is associated with risk of stress or depression.<br><13 low well-being, should investigate for depression. |
| **SEDM** | Self-efficacy. | Participants aged 12-17. | Pre and post initiation of OS-AID system. Answers regarding pre initiation relied on recollection. | 0-100<br>Higher score is associated with higher self-efficacy. |
| **HFS-C**<br><br>**HFS-P**<br><br>**HFS-PYC** | Fear of hypoglycemia, Worry sub-scale. | HFS-C: Participants aged 6-17. HFS-P: Parents of participants aged 8-17. HFS-PYC: Parents of participants aged 2-7. | Post initiation of OS-AID system. | 0-60<br>Higher scores indicate more worry concerning episodes of hypoglycemia. |
| **PSQI** | Subjective sleep quality. | Parents. | Pre and post initiation of OS-AID system. Answers regarding pre initiation relied on recollection. | 0-21<br>≤5 is associated with good sleep quality.<br>>5 is associated with poor sleep quality. |

*Suppl. Table 1: SEDM = Self Efficacy in Diabetes Management; HFS-C = Hypoglycemia Fear Survey Children; HFS-P = Hypoglycemia Fear Survey Parents; HFS-PYC = Hypoglycemia Fear Survey Parents of Young Children; PSQI = Pittsburgh Sleep Quality Index; OS-AID = Open-Source Automated Insulin Delivery*

## Suppl. Table 2

Participant characteristics at enrollment

| | All participants<br>n = 31 | Participants with available data<br>N |
|---|---|---|
| **Number of females/males** (n) | 16/15 | |
| **Age at enrollment** (years) | | |
| *Median [IQR]* | 12 [11-14] | |
| **Age at diabetes debut** (years) | | |
| *Median [IQR]* | 9.07 [6.18-10.85] | 30 |
| **Use of OS-AID system at enrollment** (years) | | |
| *Mean ± SD* | 2.37 ± 0.86 | 28 |
| **Time from diabetes debut to initiation of OS-AID system** (years) | | |
| *Median [IQR]* | 4.78[3.25-6.88] | 27 |
| **Type of CGM**[a] (n) | | |
| *Dexcom* | 27 | 28 |
| *Libre* | 1 | |
| **Type of pump** (n) | | |
| *Omnipod* | 28 | 28 |
| **Use of link** (n) | | |
| *RileyLink* | 11 | 28 |
| *EmaLink* | 5 | |
| *OrangeLink* | 11 | |
| *No link used* | 1 | |
| **Pump and CGM prior to OS-AID system (**n) | | |
| *Pump and sensor without DIY-LOOP* | 27 | 28 |
| *Commercial closed loop pump (Tandem Control IQ or Medtronic 780G)* | 1 | |
| **OS-AID system** (n) | | |
| *Android APS* | 2 | 28 |
| *Loop iOS* | 25 | |
| *OpenAPS* | 1 | |
| **Other chronic diagnosis of participant** (n) | | |
| *No other diagnosis* | 25 | 28 |
| *Other diagnosis* | 3 | |
| **Siblings of participant** (n) | | |
| *0* | 1 | 28 |
| *1* | 11 | |
| *2* | 12 | |
| *3* | 3 | |
| *4* | 1 | |
| **Adults in household** | | |
| *2* | 25 | 28 |
| *2 or more* | 3 | |
| **Mothers age at birth** (years) | | |
| *Median [IQR]* | 32 [29-33.5] | 28 |
| **Mothers' education at birth** (n(%)) | | |
| *High School* | 1 (3%) | 28 |
| *Vocational or 2-year college* | 7 (25%) | |
| *Bachelors* | 10 (36%) | |
| *Masters* | 10 (36%) | |
| **Occupational status of mother** (n(%)) | | |
| *Full time* | 20 (71%) | 28 |
| *Part time* | 8 (29%) | |

*Suppl. Table 2: OS-AID = Open-Source Automated Insulin Delivery; CGM = Continuous Glucose Monitor*

## Suppl. Table 3

BMI and BMISDS development

| n = 21<br>females/males 10/11 | **Baseline: 6-0 months before initiation of OS-AID system** | **0-6 months after initiation of OS-AID system** | **Latest available BMI data** |
|---|---|---|---|
| **Age** (years) | | | |
| *Mean ± SD* | 11.40 ± 2.92 | 11.99 ± 2.94 | 13.15 ± 3.06 |
| **BMI**[a] | | | |
| *Mean ± SD* | 17.98 ± 2.60 | 18.53 ± 2.72 | 19.65 ± 2.64 |
| **BMISDS**[b] | | | |
| *Mean ± SD* | -0.00 ± 0.25 | 0.13 ± 0.23 | 0.28 ± 0.26 |
| ***Diff. from baseline*** | | | |
| *Mean [95%CI]* | | *0.13 [-0.06;0.33]* | *0.28 [0.07;0.50]*** |
| **Hba1c** (mmol/mol) | | | |
| *Mean ± SD* | 50.08 ± 5.80 | 47.44 ± 6.47 | 44.95 ± 12.96 |
| ***Diff. from baseline*** | | | |
| *Mean [95%CI]* | | *-2.65 [-4.86;-0.42]*** | *-5.13 [-11.90;1.64]* |

*Suppl. Table 3: ** = p-value < 0.05, [a]BMI = Body Mass Index; [b]BMISDS = body mass index standard deviation score from Danish reference chart on healthy individuals; OS-AID = Open-Source Automated Insulin Delivery*
